# Validation of the PHNs precepting experiential learning causal model for preceptors of novice public health nurses in Japan

**DOI:** 10.1002/nop2.468

**Published:** 2020-03-05

**Authors:** Taeko Shimazu

**Affiliations:** ^1^ National College of Nursing Kiyose‐shi Japan

**Keywords:** experiential learning, novice public health nurse, preceptorship, public health nurse, structural equation modelling

## Abstract

**Aims:**

The purpose of this study was to analyse the relationship among public health nurse (PHN) precepting experiential learning (PHN‐PEL) and the outcomes.

**Background:**

The government supported PHNs' systematic career development and precepting learners.

**Design:**

A nationwide questionnaire survey was conducted for PHNs who precepted novice PHNs in governmental units from 2012 to 2015.

**Methods:**

“PHN Precepting Experiential Learning Scale,” “Professional Development for Public Health Nurses” and “Awareness of Responsibility for Organization” were examined through structural equation modelling.

**Results:**

Usable responses were 378 (43.5%). Significant relationships were as follows: “Role Performance of Fostering Novice PHN” related to “Self‐development as a PHN” (β = 0.72, *p* < .001); “Role Performance of Fostering Novice PHN” related to “Sharing to Foster Novice PHN” (β = 0.52, *p* < .001) and “Sharing to Foster Novice PHN” related to “Improving Career Development Environment” (β = 0.69, *p* < .001). “Role Performance of Fostering Novice PHN” and “Self‐development as a PHN” related to “Professional Development for Public Health Nurse” (β = 0.31, *p* < .001; β = 0.29, *p* < .001). Moreover, “Improving Career Development Environment” related to “Awareness of Responsibility for Organization” (β = 0.33, *p* < .001).

## 1 INTRODUCTION

For well over a decade, public health nurses have managed increasingly complex community healthcare needs often related to an ageing society, natural and man‐made disasters and the emergence of chronic diseases (Edmonson, McCarthy, Trent‐Adams, McCain, & Marshall, 2017; Kulbok, Thatcher, Park, & Meszaros, 2012). Looking ahead, the national Japanese government has issued statements about the need for systematic public health nurse (PHN) career development (Ministry of Health, Labour, & Welfare, 2003, 2005, 2007, 2017). Towards that end, one strategy the national government suggested was strengthening the “preceptorship for novice PHN training” (Ministry of Health, Labour, & Welfare, 2007). Preceptorship training is particularly important for agencies mandating PHNs become preceptors. For example, in Japan some preceptor PHNs are voluntary, but some are assigned by rotation or by a decision of the public health agencies. As a result, their motivation may be lacking and outcomes of preceptor experiences would be weakened.

Preceptor education and support are important to foster novice PHNs and to avoid preceptor's burden. Ke, Kuo, and Hung (2017), using a systematic review, identified the evidenced‐based research of the effects of one‐to‐one nursing preceptorship on new nurses' increased the following: nursing competence (Chen, Lu, & Chen, 2001; Haggerty, Holloway, & Wilson, 2013; Komaratat & Oumtanee, 2009; Kowalski & Cross, 2010; Lin & Tsai, 2010; Tsai, Lin, Chien, & Lai, 2014), job satisfaction (Chen et al., 2001) and professional socialization (Chen et al., 2001; Kowalski & Cross, 2010). Larsen et al. (2018) developed a new graduate PHN programme using the core competencies of public health nursing with the evaluation of job satisfaction, professional socialization employee retention and competencies of novice PHNs. Previous research focused on the preceptors. Mitchell, Ridgway, and Sheeran (2018) evaluated the effectiveness of preceptor education for specialty community‐based nurses. They found community‐based nurses' confidence increase in their ability to give feedback, assess clinical skills and use the clinical assessment tool. In an intervention research on web‐delivered education, Larsen and Zahner (2011) found that the variables of self‐efficacy and preceptor role knowledge of PHN independently increased after precepting student nurses. The research participants were preceptors for nursing students as opposed to novice public health nurses. Even though there would necessarily be some differences from preceptor learning by precepting novice PHNs on their roles and expectations, the outcomes suggested that the preceptor role contains opportunity for professional growth regardless of the student status. Given public health nurses demanding schedules, it is no wonder that Saeki et al. (2009) reported that PHN preceptors felt precepting was a burden. However, preceptor education based on sound educational principles might decrease the burden reported by precepting PHNs.

Because of the need to assess and act in more complex and novel community situations, PHNs need to become competent in “reflection‐in‐action” (Schon, 1983) and doing. Dewey (1938), in developing the groundwork educational principles for experiential learning, stated that a higher creative perspective would be derived through integration of reflective thinking on experiences. Kolb (1984) extended Dewey's work stating learning was a continuous and holistic process through the interaction between individuals and environment. Considering the interactive nature of learning, the experiential learning cycle would then consist of concrete experience, reflective observation, abstract conceptualization and active experimentation (Kolb & Kolb, 2008; Kolb, 1984). Furthermore, experiential learning is an accumulated learning, which means a sequence of experiences (Dewey, 1938) that assumes a spiral of knowledge creation characterizing the experiential learning process (Kolb, 1984). It is the type of knowledge that informs and refines the quality of future experience.

Even so, a PHN preceptor's learning model for precepting novice PHNs had not been fully developed and evaluated. Therefore, this study proposed to develop a preceptor learning model that was based on experiential learning theory, because the experience of fostering novice PHN could lead to experiential learning for the preceptor. When this model is used to understand PHN's preceptor experiential learning, the potential for the preceptor to experience precepting as career development could be added to the PHN career programmes.

This study was designed to create a PHNs precepting experiential learning causal model based on the PHNs preceptor experiential learning scale (PHN‐PELS) (Shimazu, 2018). Shimazu (2018) developed four subscales by which the researcher can evaluate PHNs learning through fostering novice PHN: (a) Role Performance of Fostering Novice PHN; (b) Self‐development as a PHN; (c) Sharing to Foster Novice PHN and (d) Improving Career Development Environment. Additionally, Shimazu and Asahara (2014) had previously suggested two outcomes of preceptor's learning. The first was the “Professional Development for PHNs” as they sought to explain and demonstrate the PHN role through fostering novice PHNs, and the second was “an Awareness of Responsibility for Organization” because PHNs assisted the precepted PHN to not only make sense of the organization but also to assist the organizational members in understanding the learning process of the novice PHN. “Professional Development for PHNs” and “Awareness of Responsibility for Organization” are the required mindset for PHNs in local governments.

The aim was to clarify the existing structure of the four subscales of the PHN‐PELS (Shimazu, 2018) and its relationship with the two outcomes. Creating a causal model could provide an evidence‐based rationale for developing a PHN preceptor education programme. Moreover, it would reveal the preceptor's learning process and outcome, preceptor education, preceptor support, their career development system and the PHN environment. PHNs could decrease the burden of their preceptors' role, making it a learning opportunity, and improve their career development environment.

## 2 METHODS

### 2.1 Conceptual framework

As previously noted, Shimazu (2018) developed a public health nurses precepting experiential learning scale (PHN‐PELS) consisting of four sub‐concepts: (a) “Role Performance of Fostering Novice PHN,” indicating the competence of fostering novice PHN as a professional; “(b) Self‐development as a PHN,” which means the competence of learning specialized skills through fostering novice PHN; (c)” Sharing to Foster Novice PHN,” meaning the competence of involving organizational members with fostering novice PHN; and (d) “Improving Career Development Environment,” which means the competence of improving the organizational environment for career development. The term fostering was used to denote such actions by PHN preceptors as: encouraging, nurturing, enriching, promoting, stimulating, sponsoring, assisting and facilitating.

**Figure 1:**
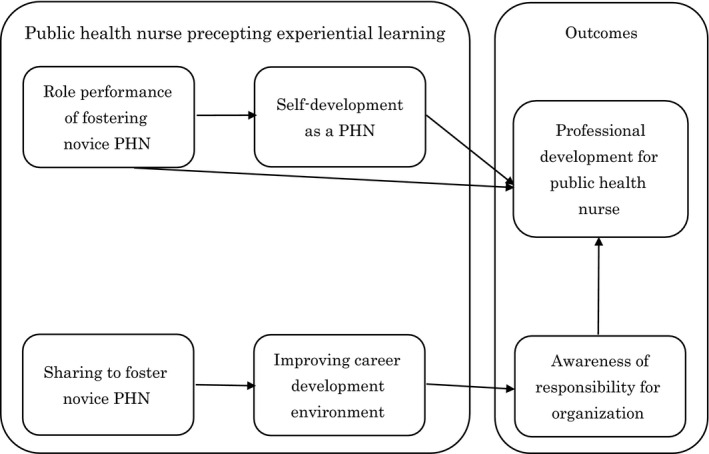
Conceptual framework of the study for the public health nurses (PHNs) precepting experiential learning causal model for preceptors of novice public health nurses

Shimazu (2018) found that the structure of public health nurses precepting experiential learning (PHN‐PEL) revealed the positive interrelationship of the four sub‐concepts and formed the conceptual framework: “Role Performance of Fostering Novice PHN” related to “Self‐development as a PHN”; “Role Performance of Fostering Novice PHN” related to “Sharing to Foster Novice PHN” and “Sharing to Foster Novice PHN” related to “Improving Career Development Environment.” PHN‐PEL had two outcomes: “Professional Development for PHN” and “Awareness of Responsibility for Organization.” Related to “Professional Development for PHN” was “Role Performance of Fostering Novice PHN” and “Self‐development as a PHN.” Moreover, related to “Awareness of Responsibility for Organization” was “Improving Career Development Environment” (Figure 1).

### 2.2 Sample and data collection procedure

This study was a nationwide cross‐sectional survey for PHNs who had any precepting experience during the years 2012–2015. Preceptors who had volunteered to precept and those who were assigned to precept were both eligible for inclusion.

The Japan is divided into 47 prefectures (administrative units similar to a state or province) consisting of 1,719 municipalities that include cities, towns, villages and 23 special wards or *ku* in Tokyo. There are 494 public health centres in Japan mainly organized in local governments such as prefectures, designated cities, special wards and other municipalities (cities, towns, villages) (Katsuda, Hinohara, Tomita, & Hamajima, 2011). The sample frame was 43 of Japan's 47 prefectures (omitted—four disaster‐stricken prefectures: Iwate, Miyagi, Fukushima and Ibaraki.): 19 of 20 ordinance‐designated cities (omitted—one disaster‐stricken city), 42 of 45 designated middle‐level cities (omitted—three disaster‐stricken cities), all seven designated cities and all 23 special wards of Tokyo. Six cities with populations over 300,000 were included because new PHNs are hired mainly at the prefecture level and large cities, not small municipalities (Japanese Nursing Association, 2015). Additionally, the author chose a scattering of 29 smaller municipalities by convenience sampling to avoid regional bias. Thus, there was a total of 163 public health centres included in the sample frame.

Invitations were sent to the 163 department heads of public health centres seeking their participation in the survey. Research and consent information and a query about the number of all PHNs having preceptor experience that fit the selection criterion were included. If the head PHNs agreed to the research, they were sent the questionnaire, which they distributed to all their PHN staff having preceptor experience. The participants responded to the questionnaire anonymously and returned it using deferred postage mail.

Those public health units agreeing to participate were 101 (59.8%): 21 of the 43 (48.8%) prefectures, 14 of the 19 (73.7%) ordinance‐designated cities, 27 of the 42 (64.3%) designated mid‐level cities, two of the seven other designated cities (28.6%), six from the 23 special wards in Tokyo (26.1%) and 31 of the 35 (88.6%) cities based on the municipalities.

Return of the questionnaire was considered as consent. The data collection was conducted from 30 November 2015 to 27 February 2016. Thus, 868 questionnaires were mailed and returned were 438 (50.5%) with 378 (43.5%) usable responses.

### 2.3 Instruments

#### 2.3.1 PHN precepting experiential learning scale (PHN‐PELS)

The PHN‐PELS was used to evaluate PHNs' learning through their experience of precepting novice PHN. This scale was developed by Shimazu (2018) and consists of four factors: (a) Role Performance of Fostering Novice PHN; (b) Self‐development as a PHN; (c) Sharing to Foster Novice PHN and (d) Improving Career Development Environment. The scale has a total of 20 Likert items with response categories ranging from 5 (*strongly agree*) to 1 (*strongly disagree*). Higher scores indicate a greater experiential learning. Shimazu (2018) confirmed the construct and content validity and the overall scale's acceptable reliability (Cronbach's alpha coefficient of .88 with sub‐factors [a] 0.76, [b] 0.80, [c] 0.71 and [d] 0.78).

#### 2.3.2 Professional development for PHN

The author used the Professional Development for PHN (PDS) developed by Okamoto, Iwamoto, Shiomi, and Kotera (2010) to evaluate outcomes of experiential learning through the experience of precepting novice PHN because it was found that precepting novice PHN increased professional development for PHN (Shimazu & Asahara, 2014). This scale consists of four factors: (a) Competency Development by Self‐responsibility; (b) Competency Development by Learning from Others/resources; (c) Succession and Improvement of One's Specialty; and (d) Behaviour According to Professional Principles. The scale has six Likert items with response categories ranging from 5 (*almost 100% agree*) to 0 (*not at all*). Higher score indicates stronger professional development. The scale has acceptable reliability (Cronbach's *α* = .93). Subscale alphas were (a) 0.89, (b) 0.85, (c) 0.85 and (d) 0.77. Moreover, it has construct validity and criterion‐related validity with correlations over .7 with two external criterions.

#### 2.3.3 Awareness of responsibility for organization

The author used the Awareness of Responsibility for Organization (ARO) developed by Suzuki (2007) to evaluate PHN's preceptors' outcome of experiential learning. Fostering novice PHN relationships with organizational members resulted in more commitment to the organization and with a broader point of view; PHNs developed their own commitment to the organization (Shimazu & Asahara, 2014; Suzuki, 2007). The scale has a total of three Likert items with response categories ranging from 5 (*strongly agree*) to 1 (*strongly disagree*). Higher scores indicate a greater awareness of responsibility for organization. Construct validity and reliability (Cronbach's alpha of .75) were confirmed.

### 2.4 Data analysis

#### 2.4.1 Item analysis

Initially, descriptive statistics were analysed. Subsequently, after checking the response bias of items for ceiling and floor effects, Pearson correlation coefficients between factors were conducted.

#### 2.4.2 Examination of PHN precepting experiential learning model

Confirmatory factor analysis (CFA) was used to examine the construct validity of each scale. Reliability was tested by Cronbach's alpha coefficient. Structural equation modelling (SEM) was used for examination of the model. The inclusion criterion for the Comparative Fit Index (CFI) was more than 0.9 and for the root mean square error of approximation (RMSEA) it was less than 0.06. SPSS Statistics version 23.0 and Amos 23.0 were used to calculate descriptive statistics and to conduct CFA and SEM.

### 2.5 Ethical considerations

Except for those four public health units continuing to struggle with the aftermath of the triple disaster (earthquake, tsunami and nuclear meltdown) in 2011 and the Ibaraki flood in 2015, each local government was invited to participate in the study. The purpose of this study was explained in writing. Participation in this study was voluntary, and consent could be retracted at any time. Returned questionnaires implied consent and were mailed back anonymously. St. Luke's International University research ethics committee approved this study (Approval No. 15‐070, 2015/11/30).

## 3 RESULTS

### 3.1 Respondent characteristics

Preceptors as respondents in this study were mainly from the larger municipalities and were women (97.6%). Their average age was approximately 39 years (Table 1). The mean duration of work experience as a PHN was 14.41 (*SD* 7.89; range 2–37) years. A large minority worked at the prefectural government level (43.9%) and at designated cities (41.8%) (Table 1).

**Table 1:** Demographics of public health nurse participants (*N* = 378)

Characteristics	*N* (%)	*M* ± *SD* (range)
Gender		
Male	9 (2.4)	
Female	369 (97.6)	
Age		38.58 ± 7.95 (24–58)
20 ~ 29	57 (15.0)	
30 ~ 39	158 (41.7)	
40 ~ 49	118 (31.1)	
50~	45 (11.9)	
Type of nursing school from which public health nurse education was obtained		
Technical nursing school	167 (44.2)	
Junior college	47 (12.4)	
University or college	159 (42.1)	
Others	5 (1.3)	
Years of experience as a public health nurse		14.41 ± 7.89 (2–37)
−5 years	50 (13.2)	
6–10 years	92 (24.3)	
11–20 years	143 (37.7)	
20 years‐	93 (24.5)	
Type of local government		
Prefecture	166(43.9)	
Designated cities	158 (41.8)	
Special wards	10 (2.6)	
Other municipalities	44 (11.6)	

Note

Prefecture: local government which organizes multiple cities or towns, villages and wards.

Designated cities: big cities which have health centre.

Special wards: big cities in Tokyo which have health centre.

Other municipalities: small cities, towns and villages which do not have health centre.

### 3.2 Item analysis of scales

Each item of the PHN‐PELS, PDS and ARO was checked by the response distribution of items (mean, *SD*, range, ceiling effects and floor effects). There were no ceiling or floor effects.

### 3.3 Relationships among PHN‐PELS subscales

According to the hypothesis, the model was adequate. Significant relationships were as follows: “Role Performance of Fostering Novice PHN” related to “Self‐development as a PHN” (β = 0.73, *p* < .001); “Role Performance of Fostering Novice PHN” related to “Sharing to Foster Novice PHN” (β = 0.63, *p* < .001) and “Sharing to Foster Novice PHN” related to “Improving Career Development Environment” (β = 0.74, *p* < .001). The goodness of fit for this model was confirmed as follows: GFI = 0.909, AGFI = 0.886, CFI = 0.913 and RMSEA = 0.057. Compared with the model of four subscales related with correlation (GFI = 0.910, AGFI = 0.884, CFI = 0.913 and RMSEA = 0.058) (Shimazu, 2018), this hypothesis model had a slightly better RMSEA and the same CFI as the previous model fit. The author adopted the hypothesis model.

### 3.4 Relationships among PHN‐PELS, PDS and ARO

Significant relationships were as follows: “Role Performance of Fostering Novice PHN” related to “Self‐development as a PHN” (β = 0.72, *p* < .001); “Role Performance of Fostering Novice PHN” related to “Sharing to Foster Novice PHN” (β = 0.52, *p* < .001) and “Sharing to Foster Novice PHN” related to “Improving Career Development Environment” (β = 0.69, *p* < .001). “Role Performance of Fostering Novice PHN” and “Self‐development as a PHN” related to “PDS” (β = 0.31, *p* < .001; β = 0.29, *p* < .001). Moreover, “Improving Career Development Environment” related to “ARO” (β = 0.33, *p* < .001) and “ARO” related to “PDS” (β = 0.39, *p* < .001).

**Figure 2:**
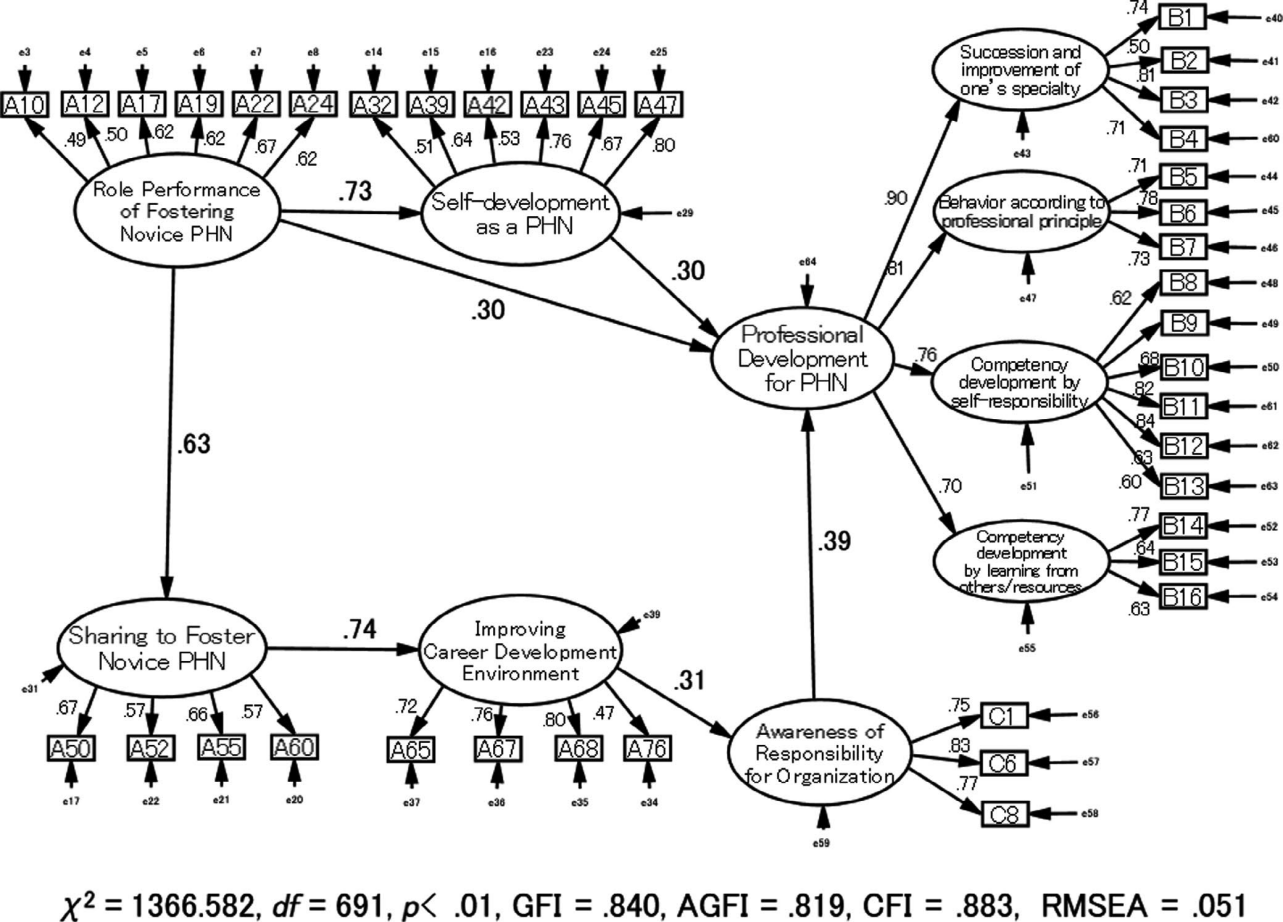
Public health nurses (PHNs) precepting experiential learning causal model for preceptors of novice public health nurses

Finally, PHN precepting experiential learning model had acceptable goodness of fit indicators as follows: GFI = 0.840, AGFI = 0.819, CFI = 0.883 and RMSEA = 0.051. These findings confirmed the goodness of fit for this model (Figure 2).

## 4 DISCUSSION

In this study, a nationwide survey provided data to develop the PHN‐PEL causal model. As a result, the author confirmed the acceptable goodness of fit for this model.

### 4.1 Participant' attributes

The participants' average PHN experience terms was almost 15 years and most were middle‐senior career. According to “Guideline for Novice Nurses Training” (Ministry of Health, Labour, & Welfare, 2011), the middle‐career PHN is a desirable preceptor; participants in this study had even longer careers. However, the results reflected the preceptor situation for PHN from the larger municipalities.

### 4.2 PHN‐PEL causal model based on experiential learning

In this study, “Role Performance of Fostering Novice PHN” related to “Self‐development as a PHN”; “Role Performance of Fostering Novice PHN” related to “Sharing to Foster Novice PHN” and “Sharing to Foster Novice PHN” related to “Improving Career Development Environment.” “Role Performance of Fostering Novice PHN” was a basis for PHN preceptor experience learning.

“Role Performance of Fostering Novice PHN” and “Self‐development as a PHN” related to “Professional Development for PHN.” This supports the previous qualitative study (Shimazu & Asahara, 2014) that suggested PHN preceptors became challenged by the need to experience both people's lives and their sense of values and that awareness needed support from various sources of information; a deepening understanding occurred through fostering novice PHN and led to the professional development for PHN.

Moreover, “Improving Career Development Environment” related to “Awareness of Responsibility for Organization.” It supports the previous study (Shimazu & Asahara, 2014) that suggested PHN preceptors acquired an awareness being a member of the organization and working with the team. “Awareness of Responsibility for Organization” includes the responsibility for the future of organization (Kanai & Suzuki, 2013, p 46), and it is important for career development to have the opportunity to improve their organizational socialization.

Furthermore, “Awareness of Responsibility for Organization” related to “Professional Development for PHN.” Swider, Levin, and Kulbok (2015) addressed PHN practice as a conceptual shift for most nurses from individual care to population‐based care. Public health science focuses on the social content and context for health at a population‐level, which is why Japanese PHNs in local governments need to become involved in policy making. When they are concerned with policy making, PHNs must also engage in teamwork and have responsibility as a member of their organization. This level of activity further engages the PHN as a professional. Because PHNs are required to follow a system and meet the needs of the community's as a whole and individual people's needs (Iwasaki, Kageyama, & Nagata, 2018), whenever PHNs experience conflicts they should work for changing the system for both the benefit of individuals and the good of the community.

The outcomes of this study contain several important implications for preceptor education and support. The first is that it provides for a conceptual awareness of precepting as a benefit to the preceptor. This could be a useful perspective for administrators who must assign precepting to PHNs and for the PHNs who receive the assignment. Then, for PHN preceptors the details about the areas where they could develop professionally are clarified so that they could fully engage in their own learning process. Preceptors would be motivated and could recognize how they improve their competence through the precepting experience. Finally, organization members would have more motivation to offer the appropriate support. For example, the importance of considering peoples' lives and their sense of values through fostering novice PHN with organizational members would be included in their education.

### 4.3 Limitations and future research

The questionnaire in this study asked about the preceptor experience in the past four years. Learning through the preceptor experience could be influenced by later events; further research is needed to examine more current experiences. The strength of this study was that it was the first nationwide survey to determine the PHN preceptor experiential learning process and findings provide consideration about effective practice and continuous education for PHNs in Japan.

Although the response rate was 59.8% for this nationwide survey, caution should be taken in generalizing the results. The response rates were higher among individual units, yet the overall results were biased towards the larger municipalities. Therefore, the results reflected the PHN preceptor situation in more urban settings. Further surveys are needed to include the results of PHNs from the smaller municipalities and to clarify the relationships among organizational environments, preceptor training programmes, career level and other variables. Moreover, there was the possibility of non‐response bias where respondents may have differed in meaningful ways from non‐respondents. Information regarding how PHNs were selected to be preceptors was not available. The selection process could have affected PHNs declining to participate especially if they felt burdened by being assigned to precept. To improve effective practices, it is necessary to develop evidenced‐based educational programmes based on sound learning theories for precepting PHNs and to create systems to support fostering novice PHNs.

## 5 CONCLUSION

This study clarified the model of PHN‐PEL based on experiential learning theory. It suggests that the PHN preceptor role of fostering novice PHNs not only leads to “Role Performance of Fostering Novice PHN,” but also to “Self‐development as a PHN,” “Sharing to Foster Novice PHN” and “Improving Career Development Environment.” Moreover, this role leads to “Professional Development for PHN” and “Awareness of Responsibility for Organization,” as required of PHNs in local governments. This understanding should encourage PHN preceptors as they become aware of what they can learn through taking on the role as a career developer for novice public health nurses. Including these variables in preceptor, education provides PHNs with this knowledge and support.

## CONFLICT OF INTEREST

The authors have no conflicts of interest to disclose.
